# Effectiveness and Safety of SYSADOAs Used in Eastern and Western Regions for the Treatment of Knee Osteoarthritis: A Systematic Review and Meta-Analysis of Randomized Controlled Trials—SYSADOAs Are Effective and Safe for Knee OA

**DOI:** 10.3390/medicina61020331

**Published:** 2025-02-13

**Authors:** Yong-Beom Park, Jun-Ho Kim

**Affiliations:** 1 Department of Orthopedic Surgery, Chung-Ang University Gwangmyeong Hospital, Chung-Ang University College of Medicine, Seoul 14353, Republic of Korea; whybe78@cau.ac.kr; 2 Department of Orthopedic Surgery, Hallym University Sacred Heart Hospital, Hallym University, 22, Gwanpyeong-ro 170beon-gil, Dongan-gu, Anyang-si 13496, Republic of Korea

**Keywords:** symptomatic slow-acting drugs for osteoarthritis, chondroitin sulfate, glucosamine sulfate, SKCPT, SKI306X, knee osteoarthritis

## Abstract

*Background and Objective*: According to international guidelines, glucosamine and chondroitin, regarded as slow-acting drugs for osteoarthritis (SYSADOAs), have been first-line treatments for knee osteoarthritis (OA); however, their efficacies remain controversial. Additionally, the efficacies of plant extract cocktails, SKI306X, and its newer formulation, SKCPT, have not been well investigated. To evaluate the effectiveness and safety of symptomatic slow-acting drugs for osteoarthritis (SYSADOAs) in patients with knee OA. *Materials and Methods*: Electronic databases were systematically searched to identify randomized controlled trials (RCTs) assessing the effectiveness and safety of SYSADOAs, including chondroitin sulfate, glucosamine sulfate, and SKCPT/SKI306X. The outcomes included pain relief, functional improvements, and safety profiles. The outcome measurements were compared between the treatment and control groups, including placebo and non-placebo groups, within and after 3 months of follow-up. *Results*: Analysis of 21 RCTs showed significantly greater improvement in pain relief in the treatment group compared with the placebo group both within (standard mean difference [SMD], 0.38; 95% confidence interval [CI], 0.18–0.57; *p* < 0.001) and after 3 months of follow-up (SMD, 0.22; 95%CI, 0.03–0.42 *p* = 0.023). The treatment group also showed significantly greater functional improvements regardless of follow-up. Pain and functional improvement did not differ significantly between the treatment and non-placebo groups. Regarding the safety profile, the risk ratios did not differ significantly between the treatment and control groups, including the placebo and non-placebo subgroups. *Conclusions*: Glucosamine, chondroitin, and SKCPT/SKI306X improved the pain and function and were non-inferior to pharmacologic drugs for up to 12 months. These findings support the clinical use of these SYSADOAs to treat knee OA. *Level of Evidence*: Therapeutic Level II.

## 1. Introduction

Osteoarthritis (OA) is a common chronic degenerative joint disease, leading to joint pain, tenderness, limited movement, and a reduced quality of life, thereby imposing social and economic burdens. With the aging population and the global increase in obesity, the burden of OA is likely to become a major challenge for health systems worldwide [1,2,3,4,5]. OA ranks seventh among causes of disability worldwide after 70 years of age. Globally, 595 million individuals (7.6% of the global population in 2020) have OA [1]. In Korea, knee OA is common, with a prevalence of 33.3–37.3% among those aged ≥50 years [6,7,8,9,10,11,12].

Drug therapy, the most used treatment option, focuses on pain relief and improving the joint function and includes non-steroidal anti-inflammatory drugs (NSAIDs), analgesics (acetaminophen or opioids), and symptomatic slow-acting drugs for OA (SYSADOA). Several international guidelines recommend NSAIDs for the management of knee OA [13,14,15,16]; however, the dose- and duration-related risks include adverse gastrointestinal and cardiovascular events [17,18].

Drug agents classified as SYSADOAs include glucosamine sulfate (GS), chondroitin sulfate (CS), diacerein, avocado–soybean unsaponifiables, and herbal medicines, which contribute to the controversy surrounding the clinical efficacy of SYSADOAs [19,20,21]. Despite this controversy, SYSADOAs are widely used worldwide to treat knee OA. A recent meta-analysis indicated that GS and CS can be considered safe treatment options for OA; however, their efficacy and the effectiveness of SYSADOAs from Eastern regions were not assessed [22]. In the US and European countries, compounds such as GS and CS are commonly used, whereas in Korea, new herbal medications are frequently prescribed [23,24]. The use of pharmaceutical-grade GS and CS is recommended for knee OA [16,25]. SKI306X or SKCPT (JOINS^®^), currently the most widely used herb-derived medicine in Korea, is formulated from the ethanol extracts of three medicinal plants: *Clematis mandshurica*, *Trichosanthes kirilowii*, and *Prunella vulgaris* [26]. Preclinical studies and clinical trials have demonstrated the efficacy and safety of this herbal medicine [27,28,29,30,31].

Therefore, this study aimed to provide clinical evidence on the efficacy and safety of GS, CS, and SKI306X or SKCPT in patients with knee OA to help inform clinical practice in the use of SYSADOAs for knee OA. We hypothesized that GS, CS, and SKI306X or SKCPT would be more effective than placebo treatments and comparable to non-placebo treatments in providing pain relief and improving function in patients with knee OA.

## 2. Materials and Methods

### 2.1. Literature Search

This systematic review and meta-analysis adhered to the Preferred Reporting Items for Systematic Reviews and Meta-Analyses (PRISMA) guidelines [32] and were registered in the International Prospective Register of Systematic Reviews (registration no. CRD42024573081). Two independent reviewers (J-H.K. and Y-B.P.) comprehensively searched using PubMed (MEDLINE), EMBASE, and the Cochrane Library electronic databases for studies indexed from 1 January 2000 to 1 October 2024, using a predefined search strategy. The year 2000 was selected to be comprehensive while limiting reports to those reflective of contemporary practice as much as possible. The search utilized the following keywords: (“chondroitin sulfate” OR “chondroitin” OR “glucosamine” OR “glucosamine sulfate” OR “SKCPT” OR “SKI306X”) AND (“knee”) AND (“osteoarthritis”). The bibliographies of the identified studies were manually reviewed to identify additional relevant articles, with no language restrictions.

### 2.2. Study Selection

Two reviewers (J-H.K. and Y-B.P.) screened the titles and abstracts. If the abstracts did not provide sufficient data, the full-text articles were reviewed. Discrepancies were resolved by consensus. Studies were included or excluded based on the Patients, Intervention, Comparison, Outcome, and Study Design (PICOS) criteria (Table 1). This systematic review included RCTs evaluating the treatment of patients with knee OA of Kellgren–Lawrence (K–L) grade I, II, or III with established SYSADOAs [33] (CS, GS, and SKCPT or SKI306X) versus placebo or non-placebo treatments, such as NSAIDs or other SYSADOAs. Studies investigating glucosamine hydrochloride were excluded as its use is not recommended as a pharmaceutical-grade drug for knee OA by the European Society for Clinical and Economic Aspects of Osteoporosis and Osteoarthritis (ESCEO) guidelines [16].

### 2.3. Assessment of Methodological Quality

Two independent reviewers (J-H.K. and Y-B.P.) assessed the risk of bias in the included RCTs using the Cochrane Handbook for Systematic Reviews of Interventions [34]. This tool evaluates bias across several domains: selection, performance, detection, and attrition. Reviewer disagreements were resolved through discussion. Publication bias was also evaluated using funnel plots and Egger’s test unless the comparison included <10 studies [34].

### 2.4. Data Extraction

The same reviewers independently extracted data from the included studies. Disagreements were resolved through discussion. The collected data included study characteristics (author, year of publication, country, and sample size), patient characteristics (mean age, sex proportion, mean body mass index, follow-up duration, and OA grading), and management details (types of treatment or control group, daily dose, treatment duration, follow-up, and rescue medicine). The outcome measures, including pain (100-mm visual analog scale [VAS] score), function (Western Ontario McMaster University Arthritis Index [WOMAC] and Lequesne index), safety (adverse events [AEs], adverse drug reactions [ADRs], and serious adverse events [SAEs]), were recorded using a predefined data form. The ADR was considered to have a causal relationship with drugs. For missing data, we first attempted to contact the authors. In the case of failure to contact, we estimated missed values using methods outlined in the Cochrane Handbook for Systematic Reviews of Interventions [34].

### 2.5. Statistical Analysis

The primary objectives of this systematic review were to compare the effectiveness (pain relief and functional improvement) and safety of SYSADOAs (CS, GS, and SKCPT or SKI306X) and the control group (placebo or non-placebo). The follow-up periods were categorized as short-term (≤3 months) and long-term (>3 months) based on previous studies [35,36]. The clinical outcomes were assessed by comparing post-medication values with pre-medication values using the Cochrane Handbook for Systematic Reviews of Interventions formulas [34]. Feasible meta-analyses were conducted to calculate the standardized mean differences (SMDs) with 95% confidence intervals (CIs) for continuous variables and risk ratios (RRs) with 95% CIs for dichotomous variables. A qualitative description of the outcomes was provided if the meta-analysis could not be performed due to insufficient data. Heterogeneity was assessed using *I*^2^ statistics to estimate the proportion of variation due to differences between studies [37]. A random effects meta-analysis was used to pool outcomes across studies. Forest plots, constructed using RevMan version 5.4 (Copenhagen, The Cochrane Collaboration), were used to display outcomes, pooled effect estimates, and overall summary effects. Statistical significance was set at *p* < 0.05.

## 3. Results

### 3.1. Study Identification

**Figure 1:**
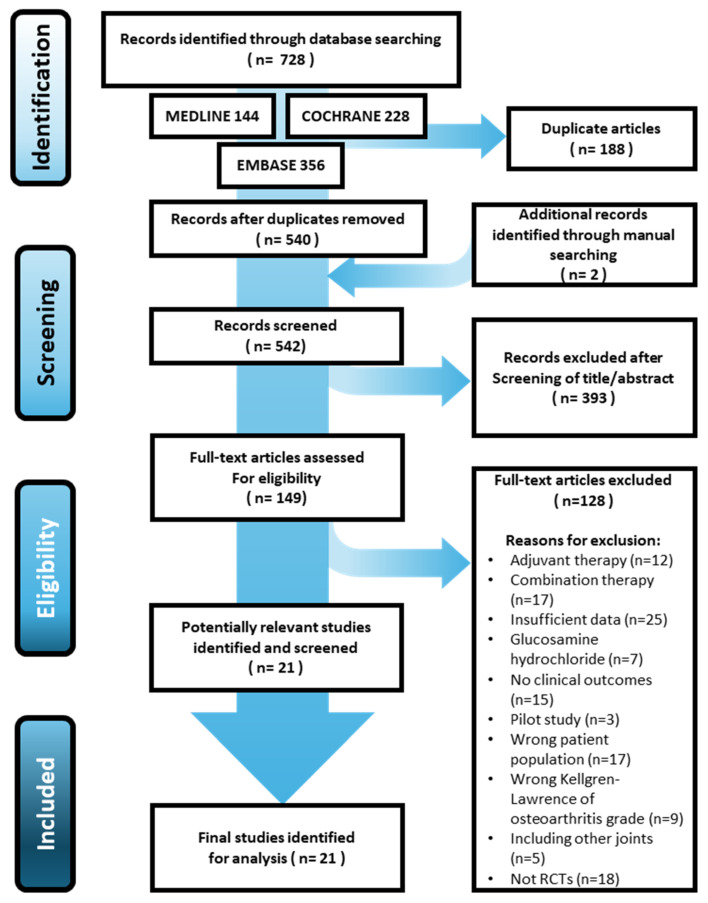
PRISMA (Preferred Reporting Items for Systematic Reviews and Meta-Analyses) flow diagram for the identification and selection of studies.

The initial electronic search identified 728 studies, with an additional two from the manual search. After removing 188 duplicates, 540 studies remained, of which 393 were excluded based on the title or abstract. An additional 128 studies were excluded after full-text review. Ultimately, 21 RCTs [26,29,31,38,39,40,41,42,43,44,45,46,47,48,49,50,51,52,53,54,55] were included in this systematic review (Figure 1).

### 3.2. Study Characteristics and Methodological Assessment

A total of 3923 knees with primary OA were included. All RCTs included patients with knee OA without K–L grade 4 [26,29,31,38,39,40,41,42,43,44,45,46,47,48,49,50,51,52,53,54,55]. Among the treatment groups in the included 21 RCTs, 11 evaluated CS [40,41,46,47,48,49,50,51,52,54,55], five evaluated GS [38,42,43,45,53], and five evaluated SKCPT or SKI306X [26,29,31,39,44] for the treatment group (Table 2). Fourteen RCTs compared to a placebo [29,31,38,41,42,43,45,46,47,48,50,52,54,55], five compared to a non-placebo such as NSAIDs [39,44,49] or SYSADOA [26,53], and two [40,51] compared CS with a placebo or non-placebo as the control group (Table 3). The risk of bias was low across most studies, with six studies [29,41,43,45,46,48] showing high-risk areas (Supplementary Figure S1). The funnel plots were symmetrical, indicating a lack of publication bias (Supplementary Figure S2). The results of Egger’s test also confirmed these trends (100-mm VAS; ≤3 months, *p* = 0.165; >3 months, *p* = 0.625).

## 4. Effectiveness

### 4.1. SYSADOAs vs. Placebo

**Figure 2:**
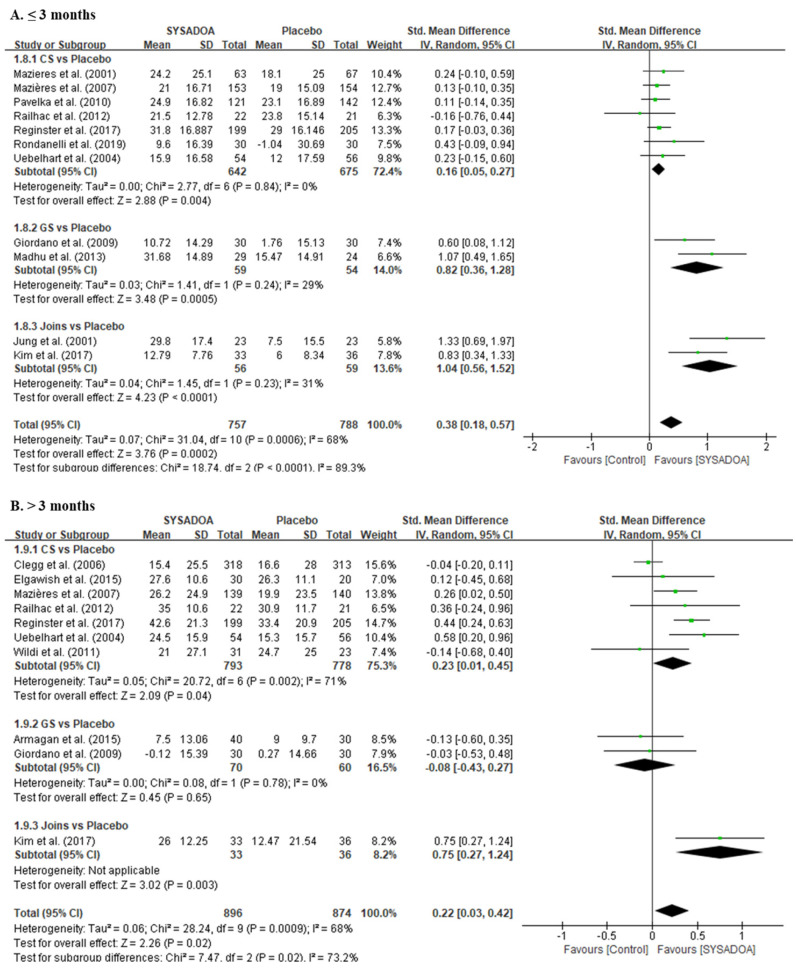
Forest plots of improvement in 100-mm VAS ≤ 3 months (**A**) and >3 months (**B**) comparing the treatment group (CS, GS, and JOINS) and the control group (placebo). Squares represent the standard mean difference in outcomes, with the size of the square being proportional to the sample size. IV, inverse variance; GS, glucosamine sulfate; CS, chondroitin sulfate; Joins, SK, SKI036X, or SKCPT; SD, standard deviation; SYSADOA, symptomatic slow-acting drugs for osteoarthritis; VAS, visual analog scale. Squares and lines represent the mean change and 95% CI in outcomes, with the size of the square being proportional to the sample size [26,29,31,38,39,40,41,42,43,44,45,46,47,48,49,50,51,52,53,54,55].

Compared with the placebo, the overall SYSADOAs showed significantly better effectiveness in the 100-mm VAS for pain relief (SMD, 0.38; 95%CI, 0.18–0.57; *p* < 0.001). The results of the subgroup analysis showed that CS, GS, and SKCPT or SKI306X all showed better effectiveness than the placebo within 3 months of follow-up. Furthermore, the treatment group showed significantly better pain relief compared with the placebo after 3 months of follow-up (SMD, 0.22; 95%CI, 0.03–0.42 *p* = 0.023) (Figure 2).

**Figure 3:**
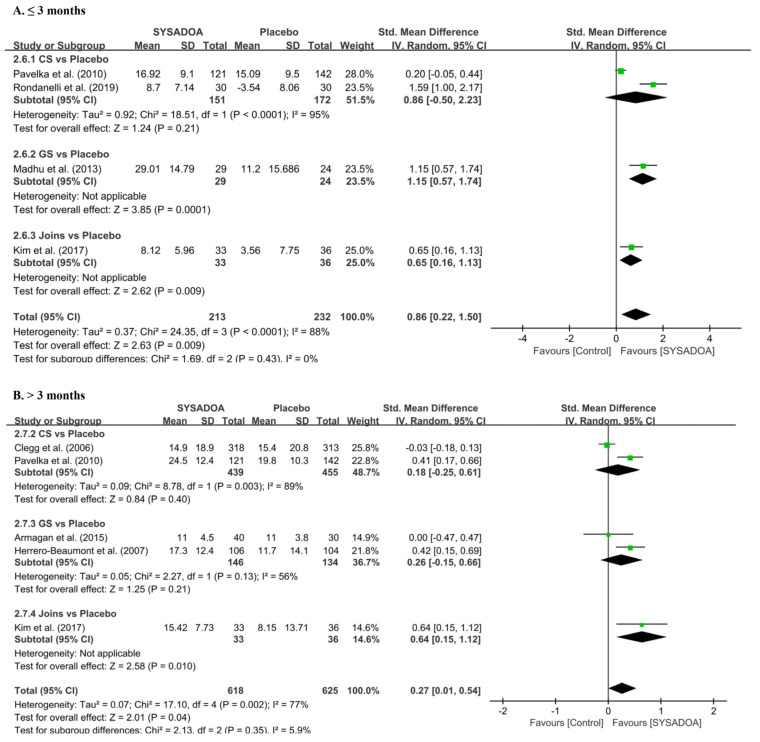
Forest plots of improvement in total WOMAC score ≤ 3 months (**A**) and >3 months (**B**) comparing the treatment group (CS, GS, and JOINS) and the control group (placebo). Squares represent the standard mean difference in outcomes, with the size of the square being proportional to the sample size. IV, inverse variance; GS, glucosamine sulfate; CS, chondroitin sulfate; Joins, SK, SKI036X, or SKCPT; SD, standard deviation; SYSADOA, symptomatic slow-acting drugs for osteoarthritis; WOMAC, Western Ontario McMaster University Arthritis Index. Squares and lines represent the mean change and 95% CI in outcomes, with the size of the square being proportional to the sample size [26,29,31,38,39,40,41,42,43,44,45,46,47,48,49,50,51,52,53,54,55].

**Figure 4:**
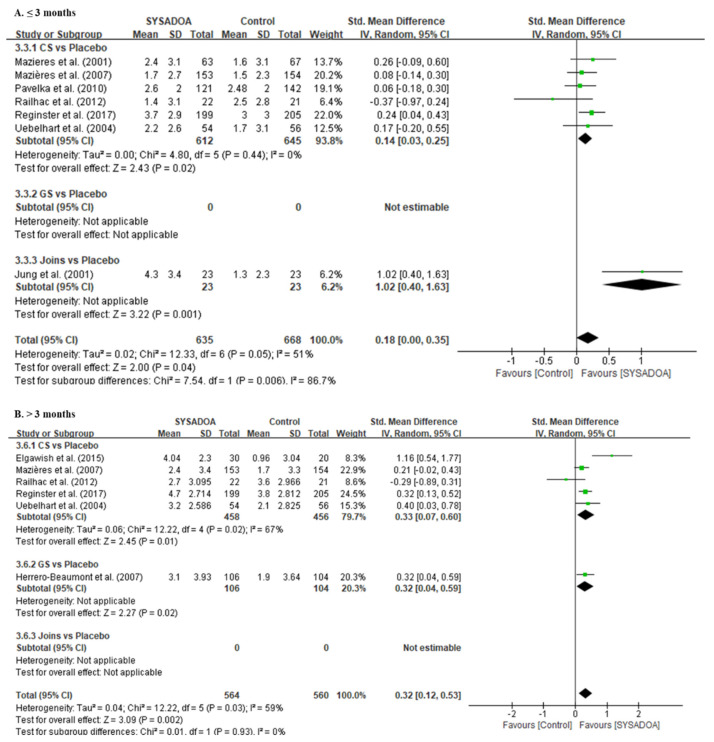
Forest plots of improvement in Lequesne index ≤ 3 months (**A**) and >3 months (**B**) comparing the treatment group (CS, GS, and JOINS) and the control group (placebo). Squares represent the standard mean difference in outcomes, with the size of the square being proportional to the sample size. IV, inverse variance; GS, glucosamine sulfate; CS, chondroitin sulfate; Joins, SK, SKI036X, or SKCPT; SD, standard deviation; SYSADOA, symptomatic slow-acting drugs for osteoarthritis. Squares and lines represent the mean change and 95% CI in outcomes, with the size of the square being proportional to the sample size [26,29,31,38,39,40,41,42,43,44,45,46,47,48,49,50,51,52,53,54,55].

Regarding functional improvement, the total WOMAC score was significantly higher in the treatment group than that in the placebo within 3 months (SMD, 0.86; 95%CI, 0.22–1.50; *p* = 0.009) and after 3 months of follow-up (SMD, 0.27; 95%CI, 0.01–0.54; *p* = 0.041) (Figure 3). Additionally, the Lequesne index was also significantly better in the treatment group than that in the placebo within 3 months (0.18; 95% CI, 0.00 to 0.35; *p* = 0.042) and after 3 months of follow-up (SMD, 0.32; 95%CI, 0.12–0.53; *p* = 0.002) (Figure 4).

### 4.2. SYSADOAs vs. Non-Placebo

Pain relief and functional improvement did not differ significantly between the treatment and the non-placebo control groups before and after 3 months of follow-up (Table 4).

## 5. Safety

No significant differences were reported in any of the safety profiles, including AEs, ADRs, and SAEs, between the treatment and control groups, including the placebo and non-placebo subgroups (Table 5).

## 6. Discussion

The principal findings of this systematic review and meta-analysis indicated that SYSADOAs, including GS, CS, and SKCPT or SKI306X, effectively improved the pain and function in knee OA. To clarify the individual effects of GS and CS, any studies involving other supplements, molecules, or combinations of GS and CS were excluded. Although the 2019 Osteoarthritis Research Society International (OARSI) guidelines did not recommend GS or CS for knee OA treatment, this study aligns with the ESCEO guidelines, which endorse GS and CS as first-line treatments for knee OA. Therefore, GS, CS, and SKCPT or SKI306X are reasonable pharmacological options for managing knee OA in clinical practice.

The results of the current meta-analysis demonstrated that the use of SYSADOAs including GS, CS, and SKI306X resulted in clinical improvement in pain for knee OA. In the short-term follow-up (≤3 months), GS, CS, and SKI306X were each associated with significant reductions in pain, as measured by the 100-mm VAS score, compared with the placebo. However, at the mid-term follow-up (>3 months), GS did not show a significant improvement compared to the placebo, while CS and SKI306X continued to show significant benefits. No significant differences were observed between SYSADOAs and non-placebo treatments in the short- or mid-term follow-up. Consistent with the overall findings of this meta-analysis, previous studies have also reported that CS and GS significantly reduce pain in knee OA [19,56,57,58]. These results suggest that SYSADOAs, including GS, CS, and SKI306X, are effective treatment options for pain management in patients with knee OA.

The results of the current meta-analysis demonstrated that SYSADOAs including GS, CS, and SKCPT or SKI306X led to functional improvement for knee OA. However, controversy persists regarding the use of GS or CS to improve function in knee OA. One meta-analysis found that while GS, CS, or their combination positively affected pain reduction, they did not significantly improve function [56]. In contrast, another meta-analysis reported that CS was effective for both pain and function, while GS was effective in narrowing the joint space. However, the combination of GS and CS showed no additional benefit [19]. Another meta-analysis concluded that the combination of GS and CS was effective and, to some extent, superior to other treatments for knee OA [58]. These heterogeneous findings may result from factors such as the concomitant use of other supplements, the administration of additional nutraceuticals, variations in GS and CS preparations, GS and CS use alone or in combination, the different stages of OA, and the varying durations of follow-up. To minimize such biases, this review excluded studies that involved other supplements or molecules, combinations of GS and CS, or patients with K–L grade 4 OA or studies without specified K–L grades. In summary, based on this meta-analysis, GS, CS, and SKCPT or SKI306X are viable options for functional improvement in patients with knee OA.

Herbal SYSADOAs are widely used in Korea, with a nationwide claims database reporting that 43.4% of patients have used one or more to treat OA [23]. SKCPT or SKI306X (JOINS^®^) is a herbal SYSADOA product formulated from a 30% ethanol dry extract of *Clematis mandshurica*, *Trichosanthes kirilowii*, and *Prunella vulgaris*, which have been widely used to treat inflammatory diseases in East Asia [39]. Preclinical studies reported positive biological effects, including anti-inflammatory actions through the suppression of pro-inflammatory cytokine expression and cartilage-protective effects via the regulation of tissue inhibitors of metalloproteinases and matrix metalloproteinase production [27,59,60]. Based on these promising findings regarding OA, clinical trials have been conducted, which have demonstrated similar pain relief and functional improvement compared with NSAIDs, but with fewer adverse events [31,39,61]. In this review, SKCPT or SKI306X demonstrated improvements in pain and function for knee OA. Therefore, this study provides robust clinical evidence supporting the prescription of SKCPT or SKI306X for the treatment of knee OA.

Adverse events did not differ significantly between the groups. In this study, adverse events did not differ significantly between GS, CS, and SKCPT and the placebo. Similarly, adverse events did not differ significantly according to the use of non-placebo treatments, including NSAIDs. This lack of difference may be attributed to the sample size in the present study, which may not have been large enough to detect subtle variations. The significant association of NSAIDs with gastrointestinal and cardiovascular complications is well established [17,62]. Discontinuing NSAIDs can reduce the risk of these adverse effects [63,64]. A recent meta-analysis reported that GS is associated with half of the adverse events compared with NSAIDs [65]. Furthermore, real-world studies have demonstrated the pharmaco-economic benefits of long-term GS, which showed a 36–50% reduction in the need for concomitant NSAID use [66]. Additionally, a previous study reported that SYSADOAs, including SKCPT or SKI306X, contributed to the discontinuation in patients with knee OA [67]. Altogether, these findings suggest that the prescription of GS, CS, and SKCPT or SKI306X is effective and safe for treating knee OA.

This study has several limitations. First, the number of studies and sample sizes were relatively small, as studies involving other supplements or molecules, combinations of GS and CS, patients with K–L grade 4 OA, and those lacking the specified K–L grades were excluded to ensure a clear assessment of effectiveness. Second, variations in the formulations, dosages of supplements, and treatment durations are potential sources of inter-study heterogeneity, which may have influenced the results. Third, not all types of SYSADOAs were included in this study. Due to the ongoing controversy regarding their efficacy, we limited our analysis to GS and CS, as recommended by the ESCEO guidelines, while excluding other agents such as diacerein and avocado–soybean unsaponifiables. This selection may limit the generalizability of our findings to other SYSADOAs. Finally, the treatment duration in most of the included studies was ≤6 months, thus limiting the ability to assess the long-term effects of these interventions.

## 7. Conclusions

The results of this study confirmed that GS, CS, and SKCPT or SKI306X improve pain and function in knee OA and are non-inferior to pharmacologic drugs for knee OA treatment over a 12-month period. These findings support their use as viable treatment options for knee OA in clinical practice.

## Figures and Tables

**Table 1:** Inclusion and exclusion criteria based on PICO ^α^.

PICO	Inclusion Criteria	Exclusion Criteria
Population	Patients with primary knee OA of K–L I, II, or III grade	- Secondary OA - Primary OA with K–L grade IV - Patients with other joints with OA, such as hip OA
Intervention	Treatment with chondroitin sulfate, glucosamine sulfate, SKI036X, or SKCPT	- Adjuvant therapy - Glucosamine hydrochloride
Comparison	Control group with placebo or non-placebo (NSAIDs or SYSADOA) treatment	- Adjuvant therapy
Outcomes	Primary outcome: pain for VAS Secondary outcomes - Function for WOMAC or Lequesne index - Safety profile including adverse events	
Study design (LOE)	I or II	III, IV or V

^α^ PICO, population intervention comparison outcome; LOE, level of evidence; OA, osteoarthritis; K–L, Kellgren–Lawrence; NSAIDs, nonsteroidal anti-inflammatory drug; SYSADOA, symptomatic slow-acting drugs for osteoarthritis; VAS, visual analog scale; WOMAC, Western Ontario McMaster University Arthritis Index.

**Table 2:** General characteristics of the included studies ^α^.

Study (Year)	Country	Tx.	Control	Sample Size ^ß^, n (Tx./Control)	Mean Age, y (Tx./Control)	Sex, M:F (Tx./Control)	Mean BMI, kg/m^2^ (Tx./Control)	K–L Grade
Armagan et al. [38] (2014)	Türkiye	GS	Placebo	40/30	56.8/55.9	30:10/25:5	30.8/31.1	II,III
Bin et al. [39] (2024)	Republic of Korea	Joins	Non-Placebo (NSAIDs)	136/142	61.1/61.7	34:102/29:113	NR	I,II,III
Clegg et al. [40] (2006)	USA	CS	Placebo Non-Placebo (NSAIDs)	318/313 318/318	58.2/58.2 58.2/59.4	113:205/131:200 113:205/106:212	32.0/31.9 32.0/31.5	II,III
Elgawish et al. [41] (2015)	Egypt	CS	Placebo	30/20	62.2/65.8	7:23/5:15	NR	II,III
Giordano et al. [42] (2009)	Italy	GS	Placebo	30/30	57.2/58.1	9:21/9:21	22/23	I,II,III
Ha et al. [26] (2016)	Republic of Korea	Joins	Non-Placebo (SYSADOA)	61/63	65.4/63.8	7:54/8:55	NR	II,III
Herrero-Beaumont et al. [43] (2007)	Spain and Portugal	GS	Placebo	106/104	63.4/64.5	10:96/15:89	27.7/27.6	II,III
Jung et al. [29] (2001)	Republic of Korea	Joins	Placebo	23/23	59.1/59.0	1:22/2:21	NR	II,III
Jung et al. [44] (2004)	Republic of Korea	Joins	Non-Placebo (NSAIDs)	125/124	60.1/59.7	9:116/9:115	NR	II,III
Kim et al. [31] (2017)	Republic of Korea	Joins	Placebo	33/36	60.2/60.0	2:31/5:31	25.6/25.6	II,III
Madhu et al. [45] (2013)	India	GS	Placebo	30/30	56.8/56.8	5:25/13:17	27.8/28.0	II,III
Mazieres et al. [46] (2001)	France	CS	Placebo	67/63	67.3/66.9	18:45/15:52	29.2/28.9	II,III
Mazieres et al. [47] (2007)	France	CS	Placebo	153/154	66/66	44:109/48:106	28.8/28.8	II,III
Pavelka et al. [48] (2010)	Czech, Hungary, Slovakia, Romania	CS	Placebo	176/181	62.2/62.3	NR	28.7/28.4	I,II,III
Pelletier et al. [49] (2016)	Canada	CS	Non-Placebo (NSAIDs)	97/97	61.4/61.3	44:53/36:61	30.1/32.3	II,III
Railhac et al. [50] (2012)	France	CS	Placebo	22/21	63.6/66.5	6:16/9:12	28.2/28.1	II,III
Reginster et al. [51] (2017)	Belgium, Czech, Italy, Poland, Switzerland	CS	Placebo Non-Placebo (NSAIDs)	199/199	65.5/65.5	43:156/39:160	30.2/29.5	I,II,III
Rondanelli et al. [52] (2019)	Italy	CS	Placebo	30/30	62.5/62.8	12:18/10:20	27.9/27.6	I,II,III
Tao et al. [53] (2009)	China	GS	Non-Placebo (SYSADOA)	45/45	64.0/62.1	20:25/18:27	NR	II,III
Uebelhart et al. [54] (2004)	USA Belgium France Switzerland	CS	Placebo	54/56	63.2/63.7	11:43/10:46	NR	I,II,III
Wildi et al. [55] (2011)	Canada	CS	Placebo	35/34	59.7/64.9	14:21/14:20	30.4/31.5	II,III

^α^ Tx., treatment; M, male; F, female; BMI, body mass index; K–L, Kellgren–Lawrence; GS, glucosamine sulfate; CS, chondroitin sulfate; Joins, SK, SKI036X, or SKCPT; NR, not reported; NSAIDs, nonsteroidal anti-inflammatory drug; SYSADOA, symptomatic slow-acting drugs for osteoarthritis. ^ß^ Sample size was reported based on the intention-to-treat set.

**Table 3:** Detailed protocol of management for knee osteoarthritis in the included studies ^α^.

Study (Year)	Daily Dose	Treatment Duration	Follow-Up, Months	Rescue Medicine
Armagan et al. [38] (2014)	Tx.: GS, 1500 mg	6 months	6	Not allowed
Bin et al. [39] (2024)	Tx.: Joins, 600 mg (2 × 300 mg) Control: Celecoxib, 200 mg (1 × 200 mg)	3 months	1, 2, 3	AAP (Max. 2 g/day)
Clegg et al. [40] (2006)	Tx.: CS, 1200 mg (3 × 400 mg) Control: Celecoxib, 200 mg (1 × 200 mg)	6 months	1, 2, 4, 6	AAP (Max. 4 g/day)
Elgawish et al. [41] (2015)	Tx.: CS, 1000 mg (1000 mg × 1)	6 months	6	AAP (Max. 4 g/day) or NSAIDs
Giordano et al. [42] (2009)	Tx.: GS, 1500 mg (1 × 1500 mg)	3 months	1, 2, 3, 4, 5, 6	AAP (0.5 g/day) or NSAIDs
Ha et al. [26] (2016)	Tx.: Joins, 600 mg (3 × 200 mg) Control: Layla (PG201), 600 mg (2 × 300 mg)	3 months	1, 2, 3	AAP (Max. 3.9 g/day)
Herrero-Beaumont et al. [43] (2007)	Tx.: GS, 1500 mg (1 × 1500 mg)	6 months	1, 3, 6	AAP (Max. 3 g/day) or NSAIDs
Jung et al. [29] (2001)	Tx.: Joins, 600 mg (3 × 200 mg)	1 month	0.5, 1	Not allowed
Jung et al. [44] (2004)	Tx.: Joins, 600 mg (3 × 200 mg) Control: Diclofenac SR 100 mg (1 × 100 mg)	1 month	1	Not allowed
Kim et al. [31] (2017)	Tx.: Joins, 600 mg (3 × 200 mg)	12 months	3, 6, 12	AAP (Max. 4 g/day)
Madhu et al. [45] (2013)	Tx.: GS, 1500 mg (2 × 700 mg)	6 weeks	3, 6 weeks	AAP (Max. 4 g/day)
Mazieres et al. [46] (2001)	Tx.: CS, 2000 mg (2 × 1000 mg)	3 months	1, 2, 3, 4, 5, 6	AAP (Max. 3 g/day)
Mazieres et al. [47] (2007)	Tx.: CS, 1000 mg (2 × 500 mg)	6 months	1, 3, 6, 8	AAP (Max. 4 g/day) or NSAIDs
Pavelka et al. [48] (2010)	Tx.: CS, 1200 mg (3 × 400 mg)	6 months	1, 2, 3, 6, 8	Allowed
Pelletier et al. [49] (2016)	Tx.: CS, 1200 mg (3 × 400 mg) Control: Celecoxib, 200 mg (1 × 200 mg)	24 months	3, 6, 12, 18, 24	AAP (Max. 3 g/day)
Railhac et al. [50] (2012)	Tx.: CS, 1000 mg (2 × 500 mg)	12 months	1, 3, 6, 9, 12	AAP (Max. 4 g/day) or NSAIDs
Reginster et al. [51] (2017)	Tx.: CS, 800 mg (1 × 800 mg) Control: Celecoxib, 200 mg (1 × 200 mg)	6 months	1, 2, 3, 4, 5, 6	AAP (Max. 3 g/day)
Rondanelli et al. [52] (2019)	Tx.: CS, 600 mg (1 × 600 mg)	3 months	1, 3	Not allowed
Tao et al. [53] (2009)	Tx.: GS, 1500 mg (3 × 500 mg) Control: Gubitong Recipe, 400 mL (2 × 200 mL)	2 months	2	NSAIDs
Uebelhart et al. [54] (2004)	Tx.: CS, 800 mg (1 × 800 mg)	0–3 months 6–9 months	3, 6, 9, 12	AAP (Max. 4 g/day)
Wildi et al. [55] (2011)	Tx.: CS, 800 mg (2 × 400 mg for first 6 months, 1 × 800 mg for next 6 months)	12 months	6, 12	AAP (Max. 3 g/day) or NSAIDs

^α^ Tx., treatment; GS, glucosamine sulfate; CS, chondroitin sulfate; Joins, SK, SKI036X, or SKCPT; NR, not reported; NSAIDs, nonsteroidal anti-inflammatory drug; SYSADOA, symptomatic slow-acting drugs for osteoarthritis; AAP, acetaminophen; Max., maximum; SR, sustained release.

**Table 4:** The weighted standard mean difference of clinical outcomes comparing the intervention group and the non-placebo control group ^α^.

Outcomes or Follow-Up SYSADOA vs. Non-Placebo	No. of Study	Sample Size, n		SMD (95% CI)	*I^2^*, %	*p* Value
		SYSADOA	Non-Placebo			
100-mm VAS						
≤3 months	6	599	612	−0.11 (−0.22 to 0.01)	0	0.06
>3 months	3	601	596	−0.16 (−0.52 to 0.19)	88	0.37
Total WOMAC score						
≤3 months	2	250	263	−0.83 (−2.41 to 0.76)	98	0.31
>3 months	2	402	401	−0.14 (−0.29 to 0.01)	6	0.06

^α^ SMD, standard mean difference; CI, confidential interval; SYSADOA, symptomatic slow-acting drugs for osteoarthritis; VAS, visual analog scale; WOMAC, Western Ontario McMaster University Arthritis Index.

**Table 5:** The risk ratio of safety comparing the intervention group and the control group ^α^.

Subgroup or Safety	No. of Study	Sample Size, n				Risk Ratio (95% CI)	*I*^2^, %	*p* Value
		SYSADOA		Non-Placebo				
		Event	Total	Event	Total			
SYSADOA vs. Placebo								
Adverse events	12	305	613	274	608	1.05 (0.97 to 1.15)	0	0.24
Adverse drug reaction	5	30	402	34	406	0.91 (0.45 to 1.82)	0	0.69
Serious adverse events	9	15	793	16	784	0.98 (0.48 to 1.97)	0	0.94
SYSADOA vs. Non-Placebo								
Adverse events	4	193	445	197	476	1.05 (0.81 to 1.36)	68	0.73
Adverse drug reaction	6	51	741	77	791	0.74 (0.47 to 1.17)	28	0.20
Serious adverse events	5	11	777	11	808	1.10 (0.48 to 2.53)	0	0.82

^α^ SYSADOA, symptomatic slow-acting drugs for osteoarthritis; CI, confidence interval.

## Data Availability

The data analyzed in this study were obtained from previously published studies and are, therefore, not available as a single dataset. Individual study data can be accessed through the cited publications.

## Supplementary Materials

The following supporting information can be downloaded at: https://www.mdpi.com/article/10.3390/medicina61020331/s1, Figure S1: The risk of bias assessment of the included studies involves a risk of bias graph (A) and summary (B); Figure S2: Funnel plots show relatively symmetric data on the 100-mm VAS within 3 months (A) and after 3 months (B) between the treatment and the placebo groups. VAS, visual analog scale.
