# Supplementary figures and images for: Effectiveness and Safety of SYSADOAs Used in Eastern and Western Regions for the Treatment of Knee Osteoarthritis: A Systematic Review and Meta-Analysis of Randomized Controlled Trials—SYSADOAs Are Effective and Safe for Knee OA
Source: Medicina (Kaunas). 2025 Feb 13;61(2):331. doi: 10.3390/medicina61020331 (PMC11857085; doi:10.3390/medicina61020331)

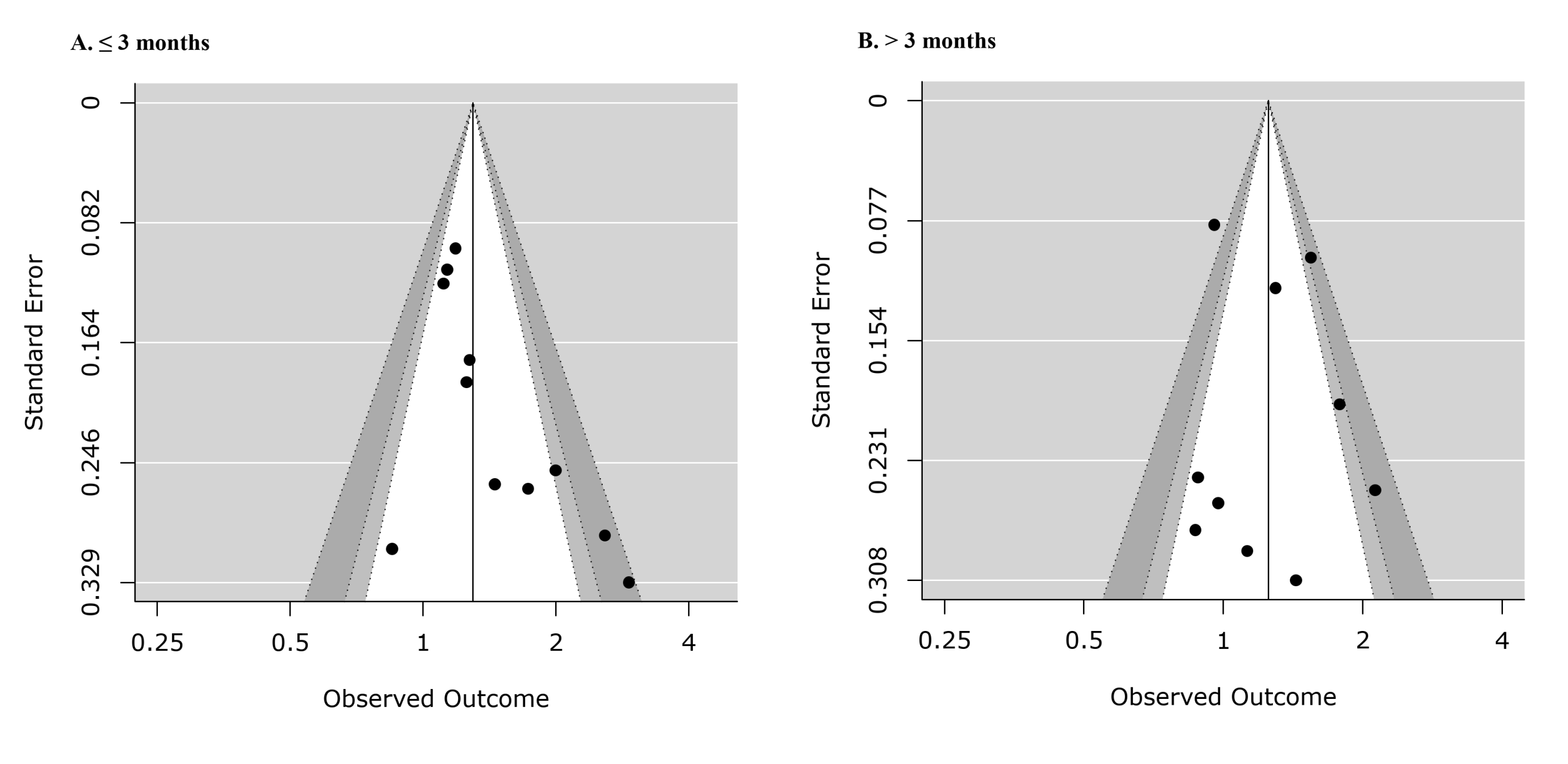

Supplement: Supplementary file 1 [file medicina-61-00331-s001.zip › Supplementary Figure S2.tif]
